# SIO-ASCO guideline on integrative medicine for cancer pain management: implications for racial and ethnic pain disparities

**DOI:** 10.1093/jncics/pkad042

**Published:** 2023-06-12

**Authors:** Kevin T Liou, Rebecca Ashare, Brooke Worster, Katie F Jones, Katherine A Yeager, Amanda M Acevedo, Rebecca Ferrer, Salimah H Meghani

**Affiliations:** Integrative Medicine Service, Department of Medicine, Memorial Sloan Kettering Cancer Center, New York, NY, USA; Department of Psychology, State University of New York at Buffalo, Buffalo, NY, USA; Department of Medical Oncology, Sidney Kimmel Cancer Center, Thomas Jefferson University, Philadelphia, PA, USA; Center for Aging and Serious Illness, Massachusetts General Hospital, Boston, MA, USA; Nell Hodgson Woodruff School of Nursing, Emory University, Atlanta, GA, USA; Behavioral Research Program, Division of Cancer Control and Population Sciences, National Cancer Institute, Rockville, MD, USA; Behavioral Research Program, Division of Cancer Control and Population Sciences, National Cancer Institute, Rockville, MD, USA; Department of Biobehavioral Health Science, School of Nursing, University of Pennsylvania, Philadelphia, PA, USA

## Abstract

Racial and ethnic disparities in pain management pose major challenges to equitable cancer care delivery. These disparities are driven by complex interactions between patient-, provider-, and system-related factors that resist reductionistic solutions and require innovative, holistic approaches. On September 19, 2022, the Society for Integrative Oncology and the American Society of Clinical Oncology published a joint guideline to provide evidence-based recommendations on integrative medicine for cancer pain management. Integrative medicine, which combines conventional treatments with complementary modalities from cultures and traditions around the world, are uniquely equipped to resonate with diverse cancer populations and fill existing gaps in pain management. Although some complementary modalities, such as music therapy and yoga, lack sufficient evidence to make a specific recommendation, other modalities, such as acupuncture, massage, and hypnosis, demonstrated an intermediate level of evidence, resulting in moderate strength recommendations for their use in cancer pain management. However, several factors may hinder real-world implementation of the Society for Integrative Oncology and the American Society of Clinical Oncology guideline and must be addressed to ensure equitable pain management for all communities. These barriers include, but are not limited to, the lack of insurance coverage for many complementary therapies, the limited diversity and availability of complementary therapy providers, the negative social norms surrounding complementary therapies, the underrepresentation of racial and ethnic subgroups in the clinical research of complementary therapies, and the paucity of culturally attuned interventions tailored to diverse individuals. This commentary examines both the challenges and the opportunities for addressing racial and ethnic disparities in cancer pain management through integrative medicine.

Pain is a prevalent symptom associated with poor quality of life, impaired physical functioning, and worse health-related outcomes in the cancer population (1). More than one-third of cancer patients remain inadequately treated for pain (2,3), and racial and ethnic disparities in pain management persist (4,5). These disparities present unique challenges because of their multifactorial nature and the complex interactions between patient-, provider-, and system-related factors that resist reductionistic solutions (6). Novel holistic approaches are required to make progress toward equitable cancer pain management.

In its Strategic Plan 2021-2025, the National Center for Complementary and Integrative Health (NCCIH) identified the research of complementary and integrative health (CIH) approaches to pain management as one of its top scientific priorities, with an emphasis on underserved racial and ethnic populations to address pain disparities (7). The term *complementary* refers to nonmainstream approaches used “together with” conventional medicine and is different than *alternative*, which refers to nonmainstream approaches used “in place of” conventional medicine. *Integrative* is a broader term that refers to the combined use of conventional and complementary approaches “in a coordinated way.” Because of their multicultural roots and origins, complementary modalities have the potential to resonate with racially and ethnically diverse populations from around the world (8). Therefore, integrative approaches, which combine conventional medicine with complementary modalities from diverse cultures, are uniquely equipped to fill gaps in cancer pain management and reduce racial and ethnic treatment disparities.

On September 19, 2022, the Society for Integrative Oncology (SIO) and the American Society of Clinical Oncology (ASCO) published a joint guideline to provide evidence-based recommendations on CIH approaches to cancer pain management (9). The guideline has the potential to expand nonpharmacologic treatment options and inform culturally attuned approaches to cancer pain management; however, key issues must be tackled to ensure equitable implementation of the guideline in diverse communities. This commentary seeks to examine the challenges and opportunities for addressing racial and ethnic pain treatment disparities in oncology through the CIH approaches described in the SIO-ASCO guideline (Box 1).

## The SIO-ASCO guideline on integrative medicine for pain management in oncology

In recent decades, CIH approaches have demonstrated a growing evidence base for pain management in cancer populations that helped inform the SIO-ASCO guideline (10-12). The NCCIH has historically classified CIH modalities into 5 broad categories: mind-body practices (eg, biofeedback, meditation, yoga), body-based practices (eg, chiropractic, reflexology), alternative medical systems (eg, Chinese Traditional Medicine, homeopathy), energy therapies (eg, reiki, breathing techniques), and natural products (eg, herbal and vitamin supplements). However, in its latest strategic plan, the NCCIH adopted a new framework to classify CIH modalities on the basis of their primary therapeutic input: physical, psychological, nutritional, or a combination of these inputs.

The latest SIO-ASCO guideline covers a range of psychological (eg, hypnosis, guided imagery), physical (eg, massage, reflexology), nutritional (eg, honey, glutamine), and combined (eg, music therapy, yoga, acupuncture) approaches (9). Acupuncture, massage, and hypnosis were recommended for cancer pain management, and the moderate strength of the recommendations indicate that results were consistent across good quality studies (with only few or minor exceptions) demonstrating a true net benefit of these modalities relative to harms. Other modalities, such as music therapy and yoga, lacked sufficient evidence to recommend for or against their use in pain management, suggesting that the true magnitude and direction of effect could not be discerned from the available studies.

This guideline has key implications for current and future research efforts to make advancements toward more equitable pain management in oncology. For the CIH modalities that lacked sufficient evidence for clinical recommendations, research should examine these through the lens of unmet pain management needs in marginalized or underserved populations, prioritizing the modalities that demonstrate unique potential to resonate with diverse populations and/or fill gaps in care. For the CIH approaches that demonstrated robust evidence for cancer pain management, an important research priority should be the mitigation of racial and ethnic disparities in the use of these evidence-based modalities.

## Racial and ethnic disparities in the use of CIH approaches for cancer pain management

A large body of research has examined racial and ethnic disparities in the use of CIH approaches for pain management in cancer populations (13). Although some research has found no statistically significant differences between racial and ethnic groups (14-18), most studies have documented key disparities in the use, acceptability, and/or accessibility of CIH approaches (19-27). For example, mind-body practices (eg, prayer, meditation) are commonly used among Black patients (28,29) and Hispanic patients (30), and traditional herbal remedies are common among Chinese patients (31,32). However, many of these commonly used modalities were deemed to have insufficient evidence in the SIO-ASCO guideline. By contrast, acupuncture and massage, both of which are recommended in the guideline, are generally less widely used among these minoritized racial and ethnic groups (25). The mechanisms and inequities underlying these racial and ethnic disparities remain an active area of research and represent promising targets to promote better pain management in underserved cancer populations.

## Challenges and barriers to addressing racial and ethnic pain disparities through CIH approaches

Growing research has highlighted possible mechanisms and inequities that could explain the documented disparities in CIH use by cancer patients from minoritized groups. Many CIH approaches are not covered by insurance (33-35). Acupuncture has historically cost on average $94-$103 per visit (36,37), and more recent estimates indicate that the total annual amount paid for acupuncturist visits was a mean of $1022 (38). Further, research from the past 2 decades suggests that 75% of adults who used acupuncture had no insurance coverage for acupuncture (39), and the proportion of acupuncturist visits with any insurance coverage has increased marginally in recent years (38). Given that members of underserved racial and ethnic groups are more likely to have lower income and higher unemployment rates than White individuals because of entrenched structural and systemic racism (40), the high costs of many CIH approaches, along with the limited insurance coverage (34,38,39,41), could potentially explain why minoritized patients were found to be less likely to use acupuncture and more likely to use CIH approaches that are free, such as prayer and meditation (25,28,29). Indeed, higher income has been shown to be an important predictor of CIH use (42).

Besides affordability, the availability of CIH approaches may also contribute to racial and ethnic disparities. For example, yoga and acupuncture clinics tend to be concentrated in upper-income neighborhoods and less accessible in neighborhoods with lower income levels and historically underserved groups (43). One study found that the availability of acupuncture, meditation, yoga, and Tai Chi was statistically significantly lower in community hospitals serving lower-income populations compared with community hospitals serving middle-income populations (44). Of the estimated 38 000 licensed acupuncturists in the United States, nearly 50% are located in New York, California, and Florida, whereas states with a larger (>30%) Black population, such as Mississippi and Louisiana, have fewer than 2 acupuncturists per 100 000 people (45). To improve accessibility for underserved geographic regions, many cancer centers are leveraging technology to deliver interventions remotely (46). Although some CIH approaches (eg, yoga, meditation) can be implemented virtually, other modalities with strong evidence base for pain, such as acupuncture and massage, are not well suited for remote delivery, which further reduces access.

In addition to costs and availability of CIH services, the belief systems of cancer patients play an important role in racial and ethnic disparities. Minoritized patients tend to have lower expectations of benefits, greater perceived barriers, and more negative social norms regarding CIH use, all of which are associated with a lower likelihood of using CIH approaches (14,22,47,48). These beliefs reflect differences in lived experiences, which may be shaped by structural racism and other systemic issues that disproportionately disadvantage certain persons or communities (49). An often overlooked influence in the belief systems of patients is the paucity of CIH practitioners of color or other minoritized backgrounds. Although information on the racial and ethnic characteristics of acupuncturists and other practitioners is limited, the available data suggest that CIH providers are disproportionately White (45,50). Patients of color may face more difficulties communicating and establishing rapport with White providers who do not come from similar backgrounds; indeed, emerging perspectives suggest these lower-quality patient–provider interactions could negatively shape expectations and beliefs surrounding CIH approaches and contribute to lower likelihood of seeking these therapies (51-53).

Just as limited diversity of CIH practitioners exacerbates racial and ethnic disparities, underrepresentation of minoritized communities in clinical research is also a key contributor. Lack of diverse participants is a major limitation of many trials of CIH approaches to cancer pain management (54). For example, the use of acupuncture for aromatase inhibitor–related joint pain received the strongest level of recommendation in the SIO-ASCO guideline (9). However, a large trial that contributed to the evidence base for this recommendation had limited representation from various racial and ethnic groups (5% Black, 7% Asian, and 9% Hispanic and Latino) (55). This underrepresentation is not unique to acupuncture research. In a recent Cochrane review of music therapy (81 trials with n = 5576 cancer patients), only 10% of trial participants were identified as Black and 6% as Latino, and most trials failed to report racial demographic information (56). More diverse inclusion in clinical trials of all CIH modalities is a critical next step to promoting greater equity in cancer pain management (57).

Finally, it is unlikely that a one-size-fits-all approach is sufficient to address racial and ethnic pain disparities in oncology (57). Acupuncture has a robust evidence base for cancer pain management relative to most other CIH approaches, but it may not be the optimal treatment option for all cancer patients with pain. Other modalities may have stronger cultural resonance with certain persons or communities and therefore have greater potential for acceptability and uptake in real-world settings. For example, ethnographic and phylogenetic research has identified music as a defining characteristic of humankind across various cultures and societies around the world (58,59). These findings highlight the unique potential of music therapy to appeal to diverse cancer populations; however, evidence of its effectiveness in cancer pain management remains insufficient to recommend its use. This is also true for other CIH modalities (eg, prayer) commonly used by minoritized racial and ethnic groups (9). There remains a critical need for more research into culturally attuned interventions, including optimal strategies for adapting existing evidence-based treatments for diverse cancer populations.

Taken together, the above factors—high costs and the lack of insurance coverage for many CIH approaches, the limited diversity and availability of CIH providers, the negative expectancy and social norms surrounding CIH use, the underrepresentation of various racial and ethnic groups in the clinical research of CIH, and the paucity of culturally attuned interventions tailored to diverse individuals—represent key barriers that must be addressed to promote equitable cancer pain management through the CIH approaches discussed in the SIO-ASCO guideline.

## Future directions and opportunities to address racial and ethnic pain disparities through CIH approaches

To date, most efforts to promote greater use and accessibility of CIH have focused on insurance policy and reimbursement. Driven in part by the opioid crisis, organizations such as the American Medical Association and the National Association of Attorneys General have called for broader insurance coverage of nonpharmacological therapies, such as acupuncture and other CIH approaches (60-62). These efforts, coupled with the growing evidence base of acupuncture, led to the recent decision by the Centers for Medicare and Medicaid Services to cover acupuncture for Medicare patients with chronic low back pain (63). Although this decision represents a promising step, further action is needed to translate greater insurance coverage of acupuncture into equitable and sustainable access for diverse cancer patients (64). Indeed, research suggests that minoritized cancer patients are half as willing as White patients to use acupuncture if treatments are covered by insurance (22). Aside from lack of previous experience with acupuncture because of cost, another possible explanation for this finding is that minoritized patients tend to have lower-quality health insurance and greater propensity to lose their existing insurance (65). These factors could make them hesitant to commit to insurance-covered treatments such as acupuncture, which often require multiple follow-up visits for effectiveness (66) and contribute to large out-of-pocket expenses for patients with high-deductible plans (38). Insurance policy reform is also needed for other modalities (eg, massage, hypnosis) that are currently recommended for pain management but not widely covered. Insurers and funders have historically cited the limited evidence base of CIH approaches as one of the main rationales behind lack of coverage (35,38). However, the publication of SIO-ASCO guidelines, with its updated review of the evidence, offers a timely opportunity to revisit these insurance coverage decisions. Centers for Medicare and Medicaid Services and insurers should consider expanding coverage to evidence-based CIH approaches that have demonstrated effectiveness for pain.

Given that insurance coverage is disproportionately lower among underserved groups, efforts to expand access must go beyond insurance policy and reimbursement. An active area of research is the investigation of novel care delivery models to reduce costs and increase access to CIH approaches. Group-based acupuncture, also known as community acupuncture, has a low-cost fee structure that results in lower out-of-pocket expenses for patients without relying on insurance reimbursements (67). In this model, providers treat patients simultaneously in a communal space, and appointments are often staggered only 10-15 minutes apart to facilitate scheduling and accommodate more patients; because of the higher clinical volume, group-based acupuncture clinics are typically able to provide services based on a sliding-scale fee (67). This model has demonstrated feasibility and a high level of acceptability among minoritized patients (68-71). Further, in a randomized trial of cancer patients with pain, group-based acupuncture produced greater pain relief and was delivered at lower cost compared with conventional acupuncture (72). Despite this promising evidence, the pain outcomes achieved with this care model have not been consistent across studies. In a large pragmatic trial of racially and ethnically diverse, medically underserved patients with chronic pain from noncancer causes, group-based acupuncture was not as effective as conventional acupuncture, and response rates for clinically meaningful pain reduction were lower relative to other acupuncture trials for pain (73).

Similarly, integrative medicine group visits, which include a variety of CIH approaches for pain management (ie, mindfulness-based techniques, acupressure, massage, nutrition), have also demonstrated feasibility and acceptability for diverse, low-income populations (74); however, the findings from a randomized trial demonstrated that the group visits did not provide benefit for pain relative to usual care (75). Taken together, the mixed findings of group-based delivery models highlight the need for additional research that strikes a delicate balance between promoting acceptability, accessibility, and adoption among diverse, underserved populations while preserving the effectiveness of CIH approaches in underresourced settings. Hybrid effectiveness–implementation study designs could play an important role in finding solutions (76).

Besides group-based formats, another promising care delivery model to expand access is the use of telehealth services, which expanded rapidly during the COVID-19 pandemic (77). Many CIH approaches have demonstrated the capacity for virtual delivery (78,79), and preliminary research suggests this may increase accessibility for racial and ethnic groups who live in neighborhoods that historically lacked CIH options (80,81). Indeed, a large proportion of cancer patients prefer to seek CIH services closer to home (82). However, some modalities are not suitable for virtual delivery (eg, acupuncture) and must be adapted in creative ways to allow for remote administration (eg, educating patients on self-acupressure through telehealth). Despite growing research in this area (78,80,83,84,85,86), rigorous large trials of virtually delivered CIH approaches are still lacking (87). Future studies in this area will need to be mindful of disparities in digital literacy and access to avoid exacerbating current unmet needs (88).

In addition to reducing costs through health policy and group-based or virtual delivery models, other strategies to increase access have focused on expanding the workforce of CIH providers. Although this type of strategy has not been widely implemented in oncology, similar efforts for other populations may provide a useful model. For example, in 2013, the Department of Defense established Acupuncture Training Across Clinical Settings (ATACS) to address unmet pain management needs among veterans (89). To promote scalability and access, ATACS taught Veterans Administration health-care providers how to deliver an adapted, standardized acupuncture protocol involving only points in the ears (89). Within 3 years, ATACS successfully trained more than 2700 providers. A large, randomized controlled trial (n = 360 cancer survivors) demonstrated that this auricular acupuncture protocol was inferior to conventional, full-body electro-acupuncture but more effective than usual care (90). Thus, in oncology settings where conventional acupuncture is not available, this auricular acupuncture protocol could potentially be taught to a nonacupuncturist workforce to expand nonpharmacological treatment options for cancer patients with pain. Similar standardized auricular acupuncture models (eg, National Acupuncture Detoxification Association protocol) have been implemented in other settings (eg, substance abuse, mental health, disaster relief) (91) and have demonstrated preliminary evidence of benefit for cancer patients (92). However, rigorous trials of these auricular acupuncture protocols for cancer pain management are still needed.

Any strategy targeting the workforce must also address the critical shortage of CIH practitioners from minoritized racial and ethnic groups and underserved communities. Existing educational programs in integrative medicine represent a logical starting point for establishing career development resources and mentorship structures to attract and cultivate a more diverse workforce of CIH practitioners (93). Collaborations with professional organizations, such as the Black Acupuncturist Association, the Latin American Music Therapy Network, and the Black Music Therapy Network, Inc, may also help strengthen support networks for aspiring CIH practitioners of color.

Because endorsement from community members is a key predictor of CIH use (47), the increased presence of CIH practitioners from minoritized and underserved backgrounds could potentially promote more positive social norms about CIH in their respective communities and lead to greater uptake of these therapies. Knowledge and awareness of CIH are also associated with higher likelihood of using these approaches (47), so more research is needed on other effective modes of information dissemination. One study, for example, found that minoritized patients prefer to receive information about yoga by smartphone (94). Another study of predominantly Black cancer patients showed that the majority wanted their primary oncologist to provide information about CIH approaches for managing pain and other symptoms (28). However, in interviews with providers and other clinical stakeholders at an academic cancer center, many expressed a lack of knowledge about the evidence base of CIH approaches (82). Previous estimates indicate that half of medical schools in the United States do not include curricula on CIH approaches, suggesting that most physicians may be poorly equipped to properly counsel patients about CIH approaches for pain management (95). Educating medical trainees about CIH is a potential intervention and does not necessarily require a major overhaul of medical curricula. Indeed, the guiding principles of integrative medicine (96,97)—its biopsychosocial framework, centrality of the doctor–patient relationship, emphasis on wellness and prevention, interprofessional collaboration, and cultural competence and inclusiveness—align closely with the core competencies of the modern medical curriculum (98,99). Training in CIH approaches will help prepare future oncology providers to counsel patients on evidence-based pain management options and equip them with more tools to address racial and ethnic pain disparities (100-102).

Educating patients and providers about CIH will go a long way toward increasing clinical trial participation among underrepresented patients (103,104). Inclusion of patient and caregiver stakeholders in the design and implementation of studies, including recruitment strategies, may also help improve diverse representation in clinical research (105). Because CIH approaches may be less familiar to patients, conventional recruitment and outreach methods may not be sufficient. Research has begun to examine which recruitment strategies are most effective for enrolling specific underrepresented groups for CIH research (106). Additional studies like these are needed to develop more targeted and data-driven approaches to enhancing diversity in clinical trials.

Finally, to make meaningful progress toward equitable cancer pain management, research needs to shift from a one-size-fits-all paradigm toward a broader array of culturally attuned interventions that can be tailored to individuals and communities of diverse backgrounds (57). Informed by culturally relevant traditions, such as African American churches, emerging research has investigated how to tailor music therapy interventions to address pain in Black noncancer populations (107,108). More recently, this line of investigation has been extended to Black cancer populations. In a retrospective study of Black and White hospitalized cancer patients who received music therapy during their routine inpatient care, both Black and White patients achieved clinically meaningful pain reduction of similar magnitude with music therapy (109); this stands in contrast to prior research of pain interventions that demonstrated only minimal or smaller responses among Black patients compared with their White counterparts (110,111). Furthermore, Black patients engaged more actively with music, and their providers referred them more frequently to music therapy for pain (109), suggesting that music therapy may not be associated with the same barriers and biases that impede conventional pharmacological treatments (112,113). Although pain reduction with music therapy was similar among Black and White cancer patients, this clinical outcome was achieved through varying approaches: spirituality and self-expression were more common themes in the music therapy sessions with Black patients, whereas family bonds and relaxation were more commonly documented among White patients (109). These preliminary findings can inform how to culturally tailor other CIH interventions to achieve equitable health outcomes.

At its core, integrative medicine is an inclusive paradigm that draws from a range of modalities from different healing traditions to promote patient-centered care (8,96). Future research of CIH approaches should embrace these culturally diverse roots to develop innovative solutions for reducing pain care disparities in oncology.

## Data Availability

No new data were generated or analyzed in support of this research.
